# Craniectomy for Malignant Cerebral Infarction: Prevalence and Outcomes in US Hospitals

**DOI:** 10.1371/journal.pone.0029193

**Published:** 2011-12-14

**Authors:** Brian P. Walcott, Elena V. Kuklina, Brian V. Nahed, Mary G. George, Kristopher T. Kahle, J. Marc Simard, Wael F. Asaad, Jean-Valery C. E. Coumans

**Affiliations:** 1 Department of Neurosurgery, Massachusetts General Hospital and Harvard Medical School, Boston, Massachusetts, United States of America; 2 Division for Heart Disease and Stroke Prevention, National Center for Chronic Disease Prevention and Health Promotion, Centers for Disease Control and Prevention, Atlanta, Georgia, United States of America; 3 Department of Neurosurgery, University of Maryland School of Medicine, Baltimore, Maryland, United States of America; 4 Department of Neurosurgery, Brown University School of Medicine, Providence, Rhode Island, United States of America; Charité Universitaetsmedizin Berlin, Germany

## Abstract

**Object:**

Randomized trials have demonstrated the efficacy of craniectomy for the treatment of malignant cerebral edema following ischemic stroke. We sought to determine the prevalence and outcomes related to this by using a national database.

**Methods:**

Patient discharges with ischemic stroke as the primary diagnosis undergoing craniectomy were queried from the US Nationwide Inpatient Sample from 1999 to 2008. A subpopulation of patients was identified that underwent thrombolysis. Two primary end points were examined: in-hospital mortality and discharge to home/routine care. To facilitate interpretations, adjusted prevalence was calculated from the overall prevalence and two age-specific logistic regression models. The predictive margin was then generated using a multivariate logistic regression model to estimate the probability of in-hospital mortality after adjustment for admission type, admission source, length of stay, total hospital charges, chronic comorbidities, and medical complications.

**Results:**

After excluding 71,996 patients with the diagnosis of intracranial hemorrhage and posterior intracranial circulation occlusion, we identified 4,248,955 adult hospitalizations with ischemic stroke as a primary diagnosis. The estimated rates of hospitalizations in craniectomy per 10,000 hospitalizations with ischemic stroke increased from 3.9 in 1999–2000 to 14.46 in 2007–2008 (p for linear trend<0.001). Patients 60+ years of age had in-hospital mortality of 44% while the 18–59 year old group was found to be 24%(p = 0.14). Outcomes were comparable if recombinant tissue plasminogen activator had been administered.

**Conclusions:**

Craniectomy is being increasingly performed for malignant cerebral edema following large territory cerebral ischemia. We suspect that the increase in the annual incidence of DC for malignant cerebral edema is directly related to the expanding collection of evidence in randomized trials that the operation is efficacious when performed in the correct patient population. In hospital mortality is high for all patients undergoing this procedure.

## Introduction

The surgical treatment of life-threatening, space-occupying cerebral edema following massive middle cerebral artery infarction, so-called “malignant” infarction, remains a controversial issue. Historically, there has been a reluctance to perform this operation given high rate of mortality and profound morbidity associated with survivors. Until recently, only case series and nonrandomized case control studies suggested any benefit of decompressive craniectomy (DC).[1]–[8] Several recent randomized controlled trials have demonstrated improved survival and functional outcome after DC in certain populations. [9]–[11] The findings from these randomized controlled trials are recapitulated in several recent reviews.[3] [12]–[13] We sought to identify trends in the prevalence and outcomes of DC for malignant cerebral infarction using the Nationwide Inpatient Sample (NIS), the largest all-payer representative sample of the US medical community.

## Methods

We studied the prevalence and outcomes of DC for malignant cerebral infarction from 1999 to 2008 using data obtained from the Healthcare Cost and Utilization Project, Agency for Healthcare Research and Quality (AHRQ) Rockville, MD.[14] The NIS is a hospital discharge database that represents approximately 20% of all inpatient admission to non-federal hospitals in the US. The NIS contains discharge data on 100% of discharges, an expanding, stratified random sample of 1,044 non-federal hospitals from 40 states in 2008. Detailed information on the design of the NIS is available at http://www.hcup-us.ahrq.gov. The NIS includes >100 clinical and non-clinical variables for each hospital stay. These include diagnoses, procedures, admission and discharge status, demographics, charges, and lengths of stay.

Patients registered in the NIS from 1999 to 2008 were included in the analysis. Those with ischemic stroke listed as the primary diagnosis were identified using the first listed International Classification of Disease 9th Revision clinical modifier (ICD-9 CM) diagnostic codes 433.11, 433.31, 433.81, 433.91, 434.01, 434.11, 434.91, and 436). Patients with the diagnosis of intracranial hemorrhage identified by ICD-9 CM diagnostic codes 430, 431, and 432.x were excluded. Also, patients with the diagnosis of posterior intracranial circulation occlusion (ICD-9 CM diagnostic codes 433.01 & 433.21) were excluded. Then, the core study population was established by selecting for those also undergoing craniotomy (including DC), identified by ICD-9 CM procedure codes 01.24 or 01.25. A subpopulation of the study population was created using ICD-9 CM procedure code 99.10 to identify those that received any form of thrombolysis. (As of 2008, an ICD-9 CM code exists specifically for thrombolysis given at referring, rather than admitting, hospitals. However, we specifically did not analyze this due to the absence of the code in the remainder of the study years). No variables for the timing of surgery, dimensions of bony decompression, stroke severity, or procedure laterality exist in the NIS dataset.

The unit of analysis was the hospital discharge. The rates of DC per 10,000 hospitalizations with ischemic stroke were assessed across 5 time intervals: 1999–2000, 2001−2002, 2003−2004, 2005−2006, and 2007−2008. We have chosen this time frame since the procedure code for infusion of thrombolytic agent (ICD-9-CM 99.10) was first introduced in October 1998. Orthogonal polynomial coefficients were obtained recursively by the method of Fisher and Yates to test linear trends for the two year intervals. The rates of DC per 10,000 hospitalizations with ischemic stroke were also reported between these two age groups: 18–59 and 60+ years. We focused our analysis on these two groups since age is considered a major prognostic factor of functional recovery after brain infarction in general (and malignant infarction in particular) and randomized trials have focused on patient of 60 years and younger.[15]–[16] Differences in rates of craniectomy by two age groups were compared by using chi-square tests.

Patient age, sex, primary payer (public, private, and others), type of admission (emergency, urgent, elective), admission source (emergency room, transfer from another hospital, transfer from long term care, and routine), hospital region (Northeast, Midwest, South, or West), hospital location and status (urban-teaching, urban-nonteaching, and rural), and bed size (small, medium, large), length of stay (days, continuous) and total hospital charges (US dollars, continuous) were coded in the NIS data. Hospital charges were converted to United States 2008 dollars using all-cities Consumer Price Index (http://data.bls.gov/cgi-bin/cpicalc.pl) Medical comorbidities were defined using markers described by Elixhauser et al[17] and calculated by use of the AHRQ software publically available at http://www.hcup-us.ahrq.gov/toolssoftware/comorbidity/comorbidity.jsp. We selected nine co-morbidity conditions (congestive heart failure, peripheral vascular disease, hypertension, paralysis, other neurological disorders, chronic pulmonary disease, diabetes with chronic complications, renal failure and coagulopathy) based on the significance of associations with in-hospital mortality reported in the previous studies.[18] We also identified four medical complications by searching the secondary diagnostic codes (ICD-9-CM codes) for pneumonia, deep vein thrombosis, pulmonary embolism, and acute myocardial infarction that were significant predictors of in-hospital mortality among hospitalizations with ischemic stroke in the same dataset.[19]

Two primary end points were examined: in-hospital mortality percentage and discharge to institutions other than home. To facilitate interpretations, adjusted prevalence was calculated from the overall and two age-specific logistic regression models using the PREDMARG statement in SUDAAN.[20] The adjusted percentage, also known as predictive margin, was generated using the logistic regression model to estimate the probability of in-hospital mortality or probability of home care discharge status, averaging over the distribution of the covariates among the entire weighted sample. The reported percentage estimates were adjusted for survey design as well as the covariates listed above.

## Results

**Figure 1:**
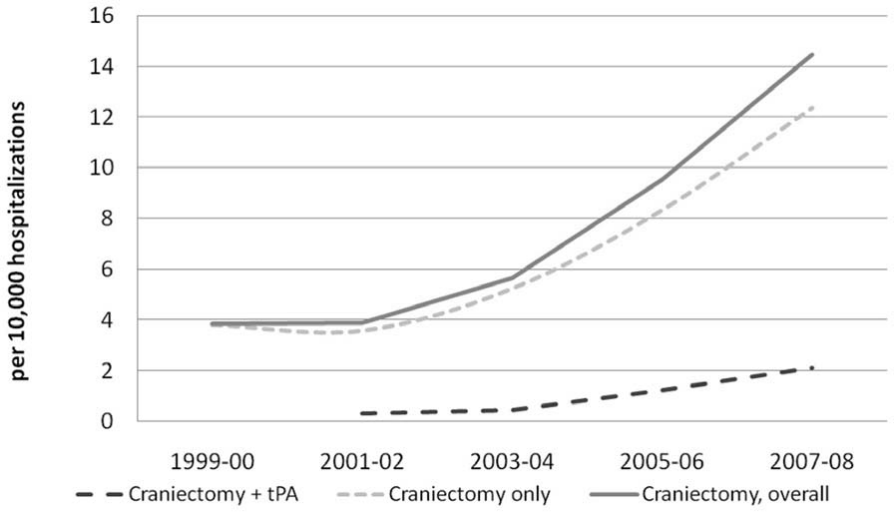
Trends in intervention procedures among hospitalizations for ischemic stroke* (n = 4,248,855). Hospitalizations with ischemic stroke listed as the primary diagnosis were identified using the first listed International Classification of Disease 9^th^ Revision clinical modifier (ICD-9 CM) diagnostic codes 433.11, 433.31, 433.81, 433.91, 434.01, 434.11, 434.91, and 436). *Patients with the diagnosis of intracranial hemorrhage (ICD-9 CM diagnostic codes 430, 431, and 432.x) and patients with the diagnosis of posterior intracranial circulation occlusion (ICD-9 CM diagnostic codes 433.01 & 433.21) were excluded. NA: estimates are not reportable due to a small sample size.

During 1999−2008 we identified 884,729 adult hospitalizations with ischemic stroke as a primary diagnosis in the NIS, representing 4,320,950 hospitalizations across the United States during this time period. (Figure 1) After excluding 71,995 patients with the diagnosis of intracranial hemorrhage and patients with the diagnosis of posterior intracranial circulation occlusion, our sample size was 4,248,955 hospitalizations. The estimated rates of hospitalizations in DC per 10,000 hospitalizations with ischemic stroke increased from 3.9 in 1999–2000 to 14.46 in 2007–2008 (p for linear trend<0.001) (Table 1). In 2007–2008, 1 out of 7 hospitalizations with DC also had performed intravenous thrombolysis with rtPA (recombinant tissue plasminogen activator). The rates of DC by two age groups are presented in table 2. The 18–59 year old group had significantly higher rates compared to 60+ year old group.

**Table 1:** Trends in intervention procedures among hospitalizations for ischemic stroke* (n = 4,248,855), Nationwide Inpatient Sample, 1999-2008.

	1999-00	2001-02	2003-04	2005-06	2007-08
	**Hospitalizations with ischemic stroke (weighted)**				
**Total hospitalizations**	909,064	883,209	830,751	803,241	822,691
**Intervention group**					
**DC + rtPA**	NR	27	36	97	174
**DC only**	346	316	435	669	1,016
**rtPA only**	8,374	8,887	10,509	16,850	24,619
**DC, overall**	351	343	471	766	1,190
	**Prevalence, per 10,000 hospitalizations with ischemic stroke**				
**DC + rtPA**	0.05 (0.00)	0.30 (0.00)	0.43 (0.00)	1.21 (0.00)	2.11 (0.00)
**DC only**	3.81 (0.01)	3.58 (0.00)	5.24 (0.01)	8.33 (0.01)	12.35 (0.01)
**rtPA only**	92.11 (0.04)	100.6 (0.05)	126.5 (0.08)	209.8 (0.10)	299.3 (0.11)
**DC, overall**	3.86 (0.01)	3.88 (0.00)	5.67 (0.01)	9.54 (0.01)	14.46 (0.01)

Hospitalizations with ischemic stroke listed as the primary diagnosis were identified using the first listed International Classification of Disease 9^th^ Revision clinical modifier (ICD-9 CM) diagnostic codes 433.11, 433.31, 433.81, 433.91, 434.01, 434.11, 434.91, and 436).

*Patients with the diagnosis of intracranial hemorrhage (ICD-9 CM diagnostic codes 430, 431, and 432.x) and patients with the diagnosis of posterior intracranial circulation occlusion (ICD-9 CM diagnostic codes 433.01 & 433.21) were excluded.

NR: estimates are not reportable due to a small sample size.

**Table 2:** Numbers and prevalence of decompressive craniectomy with and without rtPA among hospitalizations for ischemic stroke* by age (n = 4,248,955), Nationwide Inpatient Sample, 1999–2008.

Intervention group	All	18–59 years	60+ years
	**Hospitalizations with ischemic stroke (weighted)**		
**DC + rtPA**	338	237	101
**DC only**	2,783	1,784	999
**DC, overall**	3,121	2,021	1,099
			
	**Prevalence, per 10,000 hospitalizations with ischemic stroke**		
**DC + rtPA**	0.80 (0.00)	2.99 (0.00)	0.29 (0.00)
**DC only**	6.55 (0.00)	22.44 (0.01)	2.89 (0.00)
**DC, overall**	7.35 (0.00)	25.43 (0.02)	3.18 (0.00)

Hospitalizations with ischemic stroke listed as the primary diagnosis were identified using the first listed International Classification of Disease 9^th^ Revision clinical modifier (ICD-9 CM) diagnostic codes 433.11, 433.31, 433.81, 433.91, 434.01, 434.11, 434.91, and 436).

*Patients with the diagnosis of intracranial hemorrhage (ICD-9 CM diagnostic codes 430, 431, and 432.x) and patients with the diagnosis of posterior intracranial circulation occlusion (ICD-9 CM diagnostic codes 433.01 & 433.21) were excluded.

Among hospitalizations with “DC + rtPA”, in-hospital mortality was higher among 60+ years old group compared to 18–59 years old group (44% vs. 24%), although the difference did not reach statistical significance (p = 0.14) (Table 3). However, the difference was not statistically significant after adjustment for admission type, admission source, length of stay, total hospital charges, chronic comorbidities, and medical complications. Among hospitalizations with “DC only”, differences in both unadjusted and adjusted in-hospital mortality rates between two age groups were highly non-significant.

**Table 3:** In-hospital mortality and routine disposition among hospitalizations for ischemic stroke by intervention group and age (N weighted = 3,121), Nationwide Inpatient Sample, 1999–2008.

				p-value for difference 18–59 years vs. 60+
Intervention group	All	18–59 years	60+ years	
	**Hospitalizations (weighted)**			
**DC + rtPA**	338	237	101	
**DC only**	2,783	1,784	999	
**DC, overall**	3,121	2,021	1,100	
	**In-hospital mortality: Estimated percentage, %**			
	**Unadjusted**			
**DC + rtPA**	30.39 (0.06)	24.43 (0.06)	43.82 (0.12)	.14
**DC only**	23.39 (0.02)	22.63 (0.02)	24.73 (0.02)	.57
**p-value for difference DC + rtPA vs. DC only**	.23	.77	.11	
	**Adjusted: Model 1** *			
**DC + rtPA**	30.81 (0.06)	24.55 (0.06)	43.13 (0.12)	.16
**DC only**	23.56 (0.02)	22.97 (0.02)	24.75 (0.02)	.68
**p-value for difference DC + rtPA vs. DC only**	.25	.81	.13	
	**Adjusted: Model 2** *			
**DC + rtPA**	26.49 (0.04)	23.79 (0.06)	29.68 (0.07)	.56
**DC only**	23.79 (0.02)	23.00 (0.02)	25.51 (0.03)	.53
**p-value for difference DC + rtPA vs. DC only**	.56	.89	.59	
	**Routine/home care disposition: Estimated percentage, %**			
	**Unadjusted**			
**DC + rtPA**	10.31 (0.06)	15.49	0	NA
**DC only**	11.61 (0.04)	11.76	10.16	.32
**p-value for difference DC + rtPA vs. DC only**	.89	.82	NA	
	**Adjusted: Model 1** *			
**DC + rtPA**	17.11 (0.06)	24.53 (0.08)	0	NA
**DC only**	13.11 (0.01)	13.10 (0.02)	13.11 (0.03)	.99
**p-value for difference DC + rtPA vs. DC only**	.50	.16	NA	
	**Adjusted: Model 2** **			
**DC + rtPA**	19.18 (0.06)	26.53 (0.08)	0	NA
**DC only**	12.69 (0.02)	12.82 (0.02)	12.43 (0.03)	.92
**p-value for difference DC + rtPA vs. DC only**	.31	.10	NA	

*Model 1: Average Marginal Prediction: percentage estimates are adjusted for survey design as well as model covariates (gender, hospital region (Northeast, Midwest, South, or West), hospital location and status (urban-teaching, urban-nonteaching, and rural), type of admission (emergency, urgent, elective), admission status (emergency room, transfer from another hospital, transfer from long term care, and routine), payer (public, private, and others), hospital bed size (small, medium, and large), congestive heart failure, peripheral vascular disease, hypertension, paralysis, other neurological disorders, chronic pulmonary disease, diabetes with chronic complications, renal failure, coagulopathy, pneumonia, pulmonary embolism, acute myocardial infarction, and deep venous thrombosis.

**Model 2: Average Marginal Prediction: percentage estimates are adjusted for survey design as well as model covariates (all variables in the model 1 + length of stay (days, continuous) and total charges (US dollars, continuous).

Among 18–59 years old hospitalizations, no differences existed for in-hospital mortality and disposition status between “DC + rtPA” and “DC only” in both unadjusted and adjusted analysis. Among the 60+ year old hospitalization group, “DC + rtPA” group had a higher in-hospital mortalty rate (44% vs. 25%, respectively) compared to “DC only” group (although the difference did not reach statistical significance, p = 0.14). The difference became highly non-significant after adjustment for admission type, admission source, length of stay, total hospital charges, chronic comorbidities, and medical complications (30% vs. 26%, p = 0.56). In the “DC + rtPA” group, no hospitalizations with routine/home care disposition status were observed.

## Discussion

We studied a population of patients undergoing craniectomy after inpatient admission for ischemic stroke by using a nationally representative hospital discharge database. The results of our analysis showed that the annual number of craniotomies performed for this condition increased significantly and rapidly during the studied years, from 1999–2008. This large, inclusive cohort likely identifies outcomes related to actual practice patterns rather than those found in carefully selected and controlled randomized trials.

In the early modern era of neurosurgery, DC was thoughtfully employed to alleviate elevated intracranial pressure.[21]–[22] Since the first use of DC as a specific treatment for the sequelae of stroke came in 1956, many studies have demonstrated evidence for its efficacy.[23]–[26] Although these studies demonstrate increases in overall survival in selected patients, consideration of quality of life measures and functional outcomes have limited its use.[3] [27] In recent years, several randomized controlled trials were performed that demonstrated efficacy of DC for large territory cerebral stroke in patients generally less than 60 years of age.[9]–[11] There has also been evidence to reverse the longstanding dogma that dominant hemispheric stroke is associated with worse outcomes.[28]–[30] We suspect that the increase in the annual incidence of DC for malignant cerebral edema is directly related to the expanding collection of evidence in randomized trials that the operation is efficacious when performed in the correct patient population.

The pathophysiology of malignant cerebral edema is complex, but ultimately can be traced to the metabolic and hemodynamic changes that result in breakdown of the blood brain barrier. [31]–[32] Importantly, the use of rtPA in animal models has been shown to promote disruption of the blood-brain barrier that results in edema.[33] It is unclear how much the use of rtPA has to do with the development of malignant cerebral edema in humans. Patients in the subpopulation of our analysis that received rtPA had a comparable mortality following DC.[34] Confounding factors not accounted for when assessing the outcome of DC following rtPA include bias introduced from variability in stroke severity, access to care, and clinical presentation that correspond to the guidelines for safe rtPA administration.[35] In this respect, rtPA administration can be thought of as a general surrogate marker for these factors.

While in-hospital mortality tended to be higher in the 60+ age group, adjustment resulted in no identifiable difference between the two groups. We suspect that this finding relates to the higher incidence of brain herniation in this population and is heavily influenced by medical complications & comorbidities across *all* ages. The in-hospital mortality associated with surgical intervention is high in this cohort (range  = 23−43%) and comparable to previously reported randomized trials and metaanalysis when considering age group.[9]–[10] [15] While the strength of this study is its large cohort size, an inherent deficiency is that long term outcomes, quality of life, and neurological status are unable to be determined.

It should be noted that a manuscript analyzing the same topic of DC for stroke was published during the editing phase of this manuscript. Alshekhlee et al. came to a completely different conclusion, that the prevalence is the same over a similar time period. [36] While the same database was analyzed over a slightly different time period, their analysis only identified 252 patients undergoing DC (whereas our study identified 3121 patients) for ischemic stroke. These small numbers, in combination with only 502,231 patients identified as having an acute ischemic stroke, raises the possibility that the investigators did not perform a valid, accurate weighted analysis to determine true nationwide estimates.

Limitations of our analysis reflect inherent deficiencies of a nationwide, administrative database. Although coding for procedures in administrative data have been shown to be reliable, coding for medical complications and chronic co-morbidities may be prone to coding bias resulting in high specificity but low sensitivity selection.[37] Disposition status to home care must be interpreted with caution as there is no modifier available to denote situations such as comfort measures or home hospice care. There is no accounting for the timing of surgery, extent of bony decompression, stroke severity, or operative laterality in this dataset.

### Conclusion

DC is being increasingly performed for malignant cerebral edema following large territory cerebral ischemia. We ascertain that the increase in the annual incidence of DC for malignant cerebral edema is directly related to the expanding collection of evidence in randomized trials that the operation is efficacious when performed in the correct patient population. In hospital mortality is high for all patients undergoing this procedure, with the highest being in those greater than 60 years of age.
